# Validation of the Swedish Trauma Registry (SweTrau)

**DOI:** 10.1007/s00068-023-02244-6

**Published:** 2023-02-21

**Authors:** Lina Holmberg, Monica Frick Bergström, Kevin Mani, Anders Wanhainen, Håkan Andréasson, Fredrik Linder

**Affiliations:** Department of Surgical Sciences, Uppsala University, Uppsala, Sweden

**Keywords:** Validation, Trauma registry, Accuracy, Correctness, Timeliness, Comparability

## Abstract

**Purpose:**

Validation of registries is important to ensure accuracy of data and registry-based research. This is often done by comparisons of the original registry data with other sources, e.g. another registry or a re-registration of data. Founded in 2011, the Swedish Trauma Registry (SweTrau) consists of variables based on international consensus (the Utstein Template of Trauma). This project aimed to perform the first validation of SweTrau.

**Methods:**

On-site re-registration was performed on randomly selected trauma patients and compared to the registration in SweTrau. Accuracy (exact agreement), correctness (exact agreement plus data within acceptable range), comparability (similarity with other registries), data completeness (1-missing data) and case completeness (1-missing cases) were deemed as either good ($\ge$ 85%), adequate (70–84%) or poor (< 70%). Correlation was determined as either excellent ($\ge$ 0.8), strong (0.6–0.79), moderate (0.4–0.59) or weak (< 0.4).

**Results:**

The data in SweTrau had good accuracy (85.8%), correctness (89.7%) and data completeness (88.5%), as well as strong or excellent correlation (87.5%). Case completeness was 44.3%, however, for NISS > 15 case completeness was 100%. Median time to registration was 4.5 months, with 84.2% registered one year after the trauma. The comparability showed an accordance with the Utstein Template of Trauma of almost 90%.

**Conclusions:**

The validity of SweTrau is good, with high accuracy, correctness, data completeness and correlation. The data are comparable to other trauma registries using the Utstein Template of Trauma; however, timeliness and case completeness are areas of improvement.

## Background and aims

### Validation of registries

Validation of registries is a key component in ensuring solid, reliable data for research and improvement of patient care. Validations can be made in different ways, with the end goal being to ensure that the data in the registry adequately represents the population studied. Internal validity assesses whether the data in the registry is accurate when compared to an original data source (e.g. patient charts), whilst external validity evaluates whether the registry adequately captures all the population intended to be included in the registry. Validity can be assessed with *accuracy* [1–6] (the percentage of data with exact agreement between two data sets) or *correlation* [1, 2] (a correlation coefficient that also takes the possibility of chance into account). Accuracy and correlation are however not always the most suitable ways to judge a registry since there sometimes can be small deviations in data that are not clinically important but nevertheless still impact accuracy and correlation. Therefore, *correctness* [6, 7]—the sum of data with exact agreement AND data within an acceptable range—can be a more useful way to describe how well data is correct in a way that is clinically relevant. Other important aspects of validation are *case completeness*, i.e. percentage of cases that should have been included that actually are included [1, 2, 4, 6–8], as well as *data completeness* (as low percentage of missing data as possible) [1–3, 6, 7]. Finally, *comparability* [1, 2, 4]—how similar the variables of the registry are compared to other registries in the same field—and *timeliness* [1, 2, 4] (how long after the trauma the patients are registered) are also important aspects to consider.

### Severity of injuries

The goal of trauma care is to swiftly identify a severely injured patient and allocate sufficient resources at the right time. In registry-based trauma research, the definition of a severely injured patient varies [9] from any patient needing an emergency intervention within a certain time span, to a trauma patient receiving enough points on different injury scales. One of the most common ways to assess the severity of injuries is to determine the Injury Severity Score (ISS) or the New Injury Severity Score (NISS) [10]. NISS is regarded as more accurate concerning penetrating trauma [11] and in depicting in-hospital mortality [12], as well as postinjury organ failure [13]. Both ISS and NISS are calculated using the Abbreviated Injury Scale (AIS) [14], where a score of more than 15 defines a severely injured patient. The difference between ISS and NISS is that ISS is calculated using the three most severe injuries in three *different* body parts (the body is divided into 6 areas according to AIS) which are then squared and added to a sum, while NISS is calculated by squaring the three most severe injuries *regardless* of body part, which are then added to get the total sum.

### The Swedish Trauma Registry (SweTrau)

**Fig. 1:**
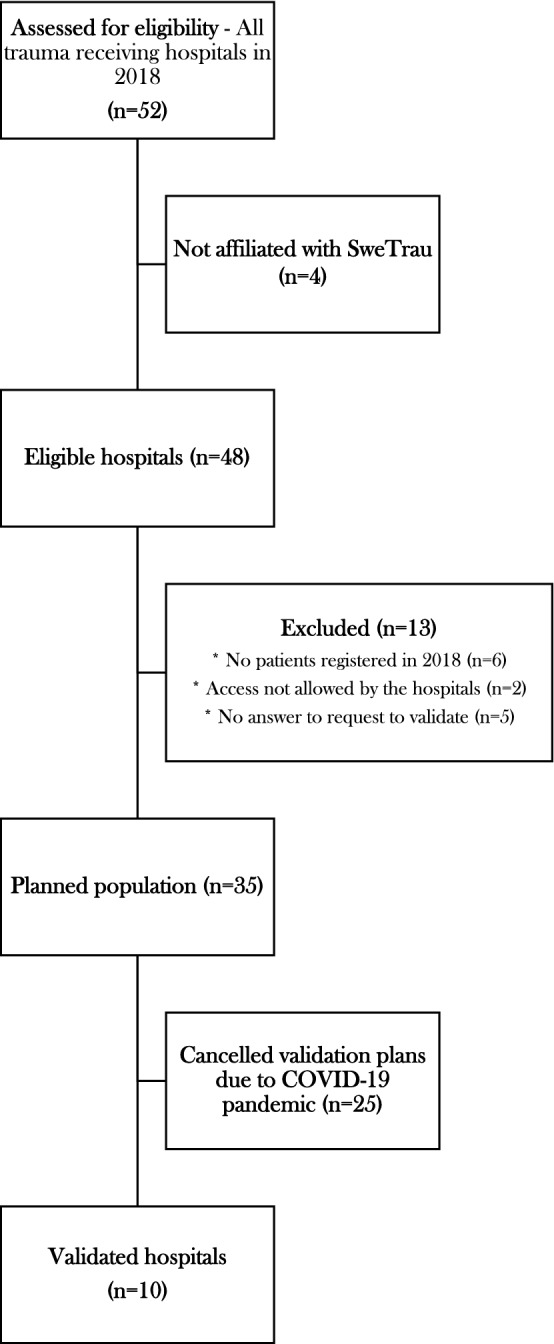
Flowchart of patient population

The Swedish Trauma Registry (SweTrau) was initiated in 2011 [15] with more hospitals gradually enrolling during the years. In 2018, 48 of 52 trauma receiving hospitals were enrolled, even though not all of them registered in SweTrau at the time (Fig. 1). The inclusion criteria of the registry are *all trauma patients that have activated a trauma call* or *admitted patients with a NISS of more than 15* or *referral patients with an NISS more than 15 and a trauma date within seven days from admission*. The exclusion criteria of SweTrau are *isolated chronic subdural haematoma* and *patients with trauma call but no trauma*. According to the Swedish National Trauma Triage Criteria (SNTTC) [16], a trauma call can be either a Trauma Alert (the highest level of trauma call) or a Trauma Response. The variables of SweTrau is based on The Utstein Template of Trauma [17].

### Trauma registries

Although there are numerous local, regional and national trauma registries existing worldwide, very few studies aimed to validate trauma registries exists, and most of the studies only validate one or two aspects of the registry. For example, in a Norwegian study of their national trauma registry [5], 144 patients were examined regarding injury coding and scoring, while in a Dutch study [18], accuracy and correlation were calculated for injury coding, scoring and survival status,. Similar, in a study from the Netherlands [19], injury coding, scoring and survival status were assessed, however, only correlation was calculated. Furthermore, trauma registries are difficult to compare due to the difference in variables. In 2011, Ringdal et al. [20] showed in a large multicenter study that a number of international trauma registries had implemented The Utstein Template of Trauma [21] for a more uniform reporting of variables in trauma, which SweTrau also uses. Other trauma registries utilizing the Utstein Template of Trauma are Major Trauma Registry of Navarre (MTRN, Spain), and the Helsinki Trauma Registry (HTR, Finland). MTRN has been evaluated in two studies for case completeness [22] as well as data completeness and correctness [23]. In a study examining all registered patients (312) in HTR during 2013 [6], accuracy, correctness, data completeness and case completeness were calculated, while correlation, timeliness and comparability were not evaluated. To our knowledge, a validation of a national trauma registry that examines all these parameters has not been performed. Our hypothesis at the outset of this study was that the data in SweTrau is valid and can be used for reliable trauma research. The data of SweTrau, however, has not yet been validated which is why we believe this study to be an important addition to international trauma research.

### Aim

The aim of this study was to validate SweTrau by assessing accuracy, correctness, correlation, data completeness, case completeness, timeliness (efficiency) and comparability.

## Materials and methods

### Re-registration process

We sought to validate SweTrau by on-site visits while manually re-registering a number of randomly selected trauma patients registered in 2018 and comparing them to the original registration in SweTrau. This was done by two of the authors (LH and MFB) who have completed the AIS course and are experienced SweTrau registrars. The re-registration was made by examining the patient charts and paramedic notes and then compare the original data with the registration in SweTrau. The intent was to validate approximately 5% of the total registrations, in line with the recommendations of American College of Surgeons [24] for validating trauma registries (*to re-abstract 5–10% of patient records*). An initial estimation of 400 patients as sample size was re-adjusted to 450 (5.1%) when the SweTrau’s annual report from 2018 was released (8862 patients registered in 2018). We planned to validate 30 patients in each of the seven university hospitals, as well as ten patients at the regional and local hospitals. Out of 52 trauma receiving hospitals, 48 were affiliated with SweTrau. Thirteen hospitals were excluded due to: not having registered any patients in SweTrau (*n* = 6), authors not allowed access to hospital records due to logistic issues (*n* = 2) and failure to answer our request to validate (*n* = 5). Thus, 35 hospitals were planned to take part in the validation process (Fig. 1). Unfortunately, due to the COVID-19 pandemic, access to hospitals for validation was severely restricted, and the study was only possible to carry out on-site re-registration at 10 different trauma receiving hospitals (120 patients) before all possibilities of validation at the included hospitals were withdrawn.

### Validation terms

*Accuracy* was determined as the percentage of data with exact agreement between the original registration and the re-registration.

*Correctness* was defined as the sum of data with exact agreement AND data within acceptable range. Data within acceptable range was defined as the following difference:

- Glasgow Coma Scale (GCS) upon arrival of EMS personnel at scene/upon arrival in hospital: ± 1 point.
- Number of days on ventilator: ± 1 day.
- Respiratory rate (RR) upon arrival of EMS personnel at scene/upon arrival in hospital: ± 5 breaths.
- Systolic blood pressure (SBP) upon arrival of EMS personnel at scene/upon arrival in hospital: ± 10 mmHg.
- Time of trauma/alarm/arrival at scene/EMS personnel leaving the scene/arrival in hospital/first CT scan/until first key emergency intervention: ± 10 min.

All other variables (e.g. ISS, NISS, intervention etc.) were only assessed with exact agreement which therefore equals the correlation.

*Correlation* of the variables in SweTrau and the re-registration was calculated with either *Cohen’s Kappa* (categorical, qualitative data) or *Pearson’s correlation coefficient* (numerical, quantitative data). The correlation was determined as either excellent ($\ge$ 0.8), strong (0.6–0.79), moderate (0.4–0.59) or weak (< 0.4).

*Data completeness* was calculated by checking the missing data in SweTrau, after adjusting for when a variable is not applicable (some variables in SweTrau are mutually exclusive, for example *Respiratory rate upon arrival in hospital* and *Respiratory rate clinical category upon arrival in hospital*).

*Case completeness* was determined by searching the hospitals’ individual emergency ledgers for two randomly selected weeks during 2018. The charts of the patients that might fulfil the inclusion criteria for SweTrau were examined and the patients that should have been registered in SweTrau were then checked to see if they had indeed been included, and the percentage was calculated.

A*ccuracy*, *correctness, data completeness* and *case completeness* were determined as either good ($\ge$ 85%), adequate (70–84%) or poor (< 70%).

*Timeliness* (efficiency) was recorded by comparing the date of trauma with the date of the signed registration in SweTrau. The earliest possible registration in SweTrau is 31 days after the trauma (unless the patient dies before that), due to the 30-day mortality variable.

*Comparability* was assessed by comparing the variables in SweTrau with the Utstein Template of Trauma [17, 21], an international consensus document regarding trauma registry variables.

### Validation of ISS and NISS

The ISS/NISS-registrations were validated in three different ways. Firstly, by comparing the exact ISS/NISS score. Secondly, by dividing the score into intervals: *ISS/NISS 0–8 (mild* *injury*), *9–15 (moderate injury)*, *16–24 (severe injury*) and > *25 (very severe injury)* as a modification of the six Copes’ categories [25, 26]. The ISS/NISS score were deemed correct if the original registration and the re-registration were scored in the same interval. Finally, by dividing and comparing the ISS/NISS score with a cut-off of *ISS/NISS 0–15 (not severe injury)* and *ISS/NISS* > *15 (severe injury)* respectively.

### Statistics

Statistical analyses were performed with IBM SPSS Statistics, version 26 (IBM Corp., Armonk, N.Y., USA), and with Microsoft Excel for Mac, version 16.16.22. Categorical data were analyzed with Chi-squared test. The level of significance was set at a *p* value less than 0.05.

## Results

Overall demographics of the study population compared to all patients in SweTrau 2018 is presented in Table 1. There was no difference between the groups. The majority of the patients were male (68.3%) and had suffered a blunt trauma (90.0%). Most of them were relatively healthy with an ASA score 1–2 (85.8%). The mean and median of age was 44 and 43 years, while the mean and median of NISS and ISS was 12 and 9 vs 9 and 5, respectively. All variables of SweTrau except the individual AIS-codes were examined, rendering 48 individual variables and four groups of variables to be validated (Table 2). None of the patients with NISS < 15 died. Two patients (1.7%) had a NISS < 15 at the initial registration but had a missed injury that led to NISS > 15 at re-registration. One patient (0.8%) was determined severely injured (NISS > 15) at the initial registration but the NISS was reduced to < 15 at the re-registration. For the rest if the patients, the NISS score was either an absolute agreement between the initial registration and the re-registration, or differed upward or downward but without affecting the limit of NISS < 15 < .

**Table 1:** Patient demographics

	Population *n* = 120	SweTrau 2018 *n* = 9389	*P* value^a^
Male sex (%)	82 (68.3)	6168 (65.7)	0.366
Blunt trauma (%)	108 (90.0)	8610 (91.7)	0.451
ASA score of 3 or higher (%)	17 (14.2)	1180 (12.6)	0.275

*ASA score* American Society of Anesthesiologists physical status score

^a^χ^2^ test

**Table 2:**
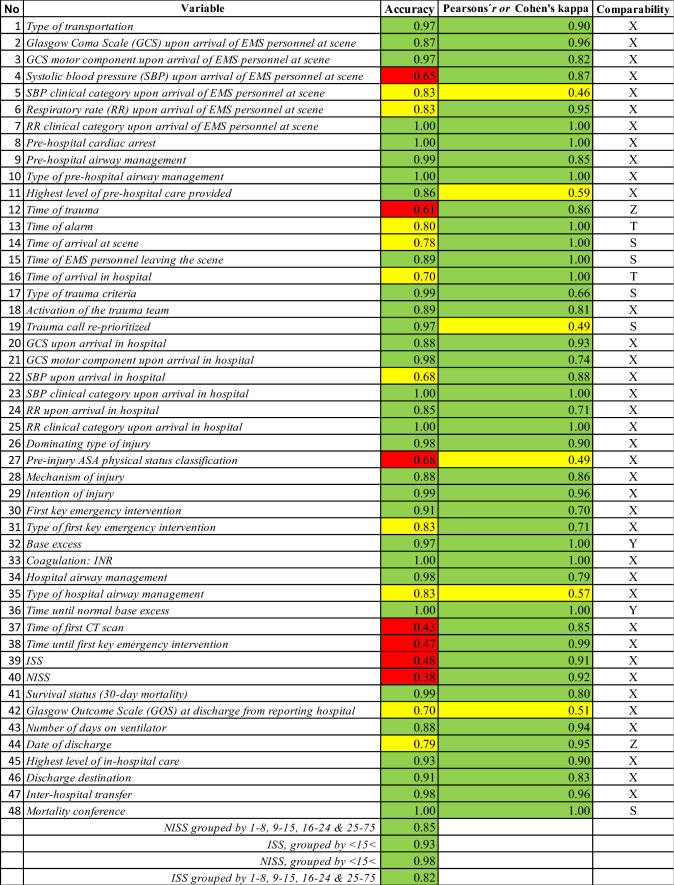
Accuracy, correlation and comparability

S = variables exclusive to SweTrau (*n* = 5). T = time of alarm and time of arrival in hospital instead of time from alarm to hospital arrival (*n* = 2). X = exact agreement (*n* = 37). Y = venous or arterial blood

### Accuracy

Overall accuracy was 85.8%. Out of 48 variables, 41 exhibited good ($\ge$ 85%, 31 variables) or adequate accuracy (70–84%, 10 variables) (Table 2). Regarding *NISS* and *ISS,* they showed good accuracy when *grouped by* < *15* < (97.5% and 92.5%), as well as *grouped by 1–8, 9–15, 16–24 and 25–75* (85.0% and 81.7%), although their individual accuracy was poor (*NISS* 38.3% and *ISS* 48.3%)*.* Five additional variables had poor accuracy (< 70%): *Time of first CT scan* (39.6%)*, Time until first key emergency intervention* (47.4%)*, Time of trauma* (60.0%), *Systolic Blood Pressure (SBP) upon arrival of EMS personnel at scene* (64.8%) and *Pre-injury ASA physical status classification* (68.1%)*.*

### Correlation

The majority of the variables (87.5%, 42 of 48 variables) had an excellent ($\ge$ 0.8) or strong correlation (0.6–0.79) between the original registration and the re-registration (Table 2). Six variables showed moderate correlation (0.4–0.59): *SBP clinical category upon arrival of EMS personnel at scene* (0.46), *Pre-injury ASA physical status classification* (0.49), *Trauma call re-prioritized* (0.49), *Glasgow Outcome Scale (GOS) at discharge from reporting hospital* (0.51), *Type of airway management* (0.57), *Highest level of pre-hospital care provided* (0.59). No variable had a weak correlation (< 0.4).

### Correctness

**Fig. 2:**
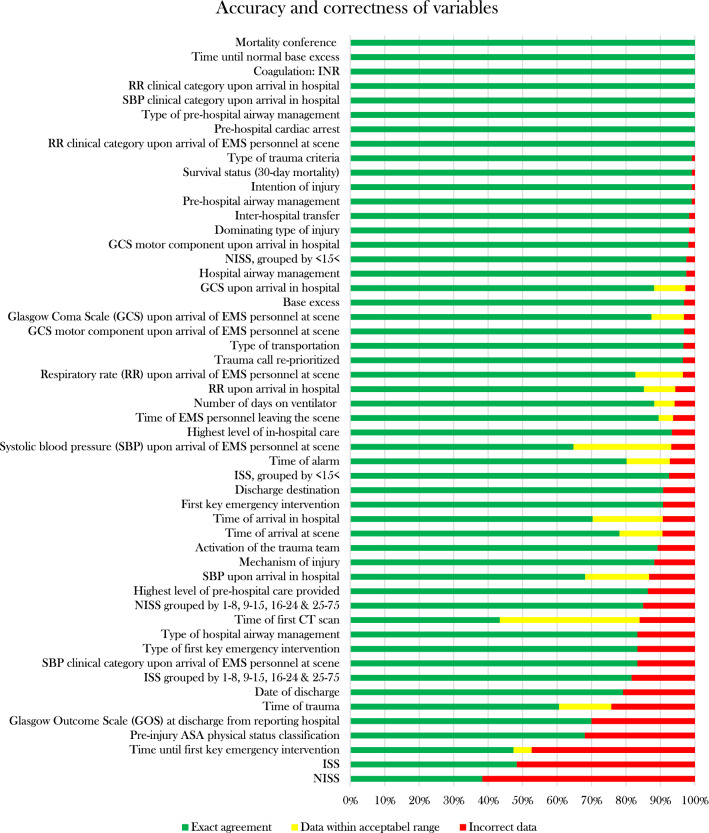
Accuracy and correctness of variables. *RR* respiratory rate, *EMS* emergency medical services, *NISS* New Injury Severity Score, *GCS* Glasgow Coma Scale, *SBP* systolic blood pressure, *GOS* Glasgow Outcome Scale

Overall correctness was 89.7%. Of the individual variables, 44 of 48 (91.7%) had a good ($\ge$ 85) or adequate (70–84%) correctness, as well as all the groups of ISS and NISS (Fig. 2)**.** Four variables exhibited poor correctness (< 70%): *NISS* (38.3%), *ISS* (48.3%), *Time until first key emergency intervention* (52.6%), *Pre-injury ASA physical status classification* (68.1%).

### Data completeness

**Fig. 3:**
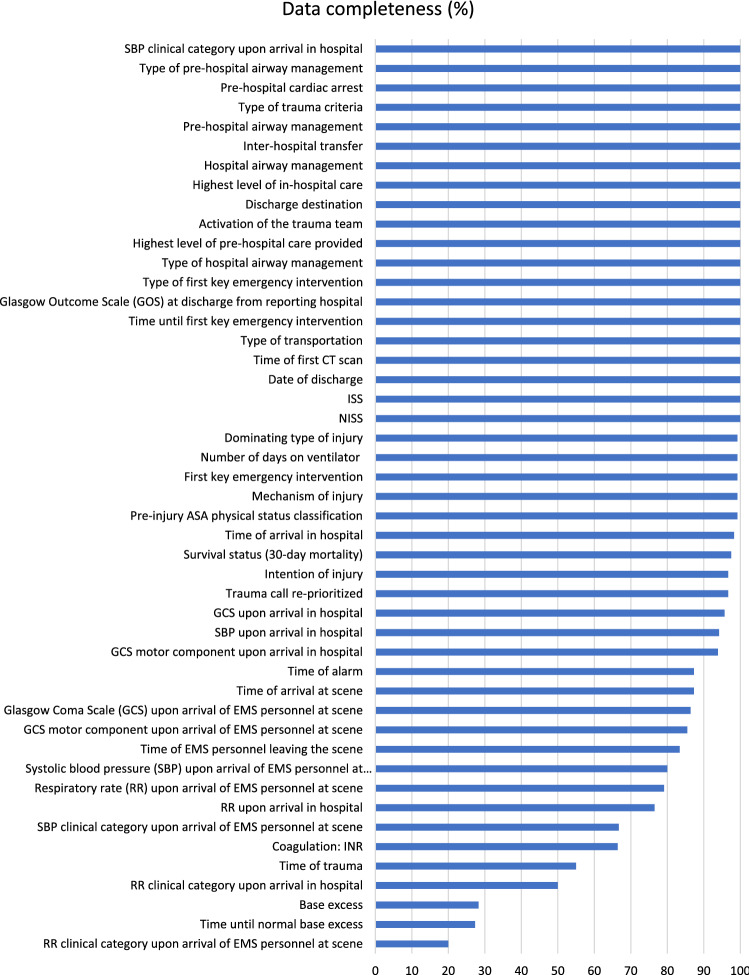
Data completeness and missing data. *SBP* systolic blood pressure, *GOS* Glasgow Coma Scale, *CT* computed tomography, *ISS* Injury Severity Score, *NISS* New Injury Severity Score, *ASA score* American Society of Anesthesiologists physical status score, *GCS* Glasgow Coma Scale, *EMS* emergency medical services, *RR* respiratory rate

Overall data completeness was 88.5%. Most variables (41/48) exhibited a good ($\ge$ 85%) or adequate (70–84%) data completeness (Fig. 3). Seven variables showed poor completeness < 70%: *RR clinical category upon arrival of EMS personnel at scene* (20.0%)*, Time until normal base excess* (27.3%)*, Base excess* (28.3%)*, RR clinical category upon arrival in hospital* (50.0%)*, Time of trauma* (55.0%)*, Coagulation: INR* (66.4%)*, SBP clinical category upon arrival of EMS personnel at scene* (66.7%)*.*

### Case completeness

A total of 44.3% of the identified trauma patients eligible for inclusion in SweTrau were registered (39/88). None of the missed patients were severely injured (NISS > 15) or had initiated the highest level of trauma call (Trauma Alert). The missed patients were evenly dispersed among the validated hospitals.

### Timelines

**Fig. 4:**
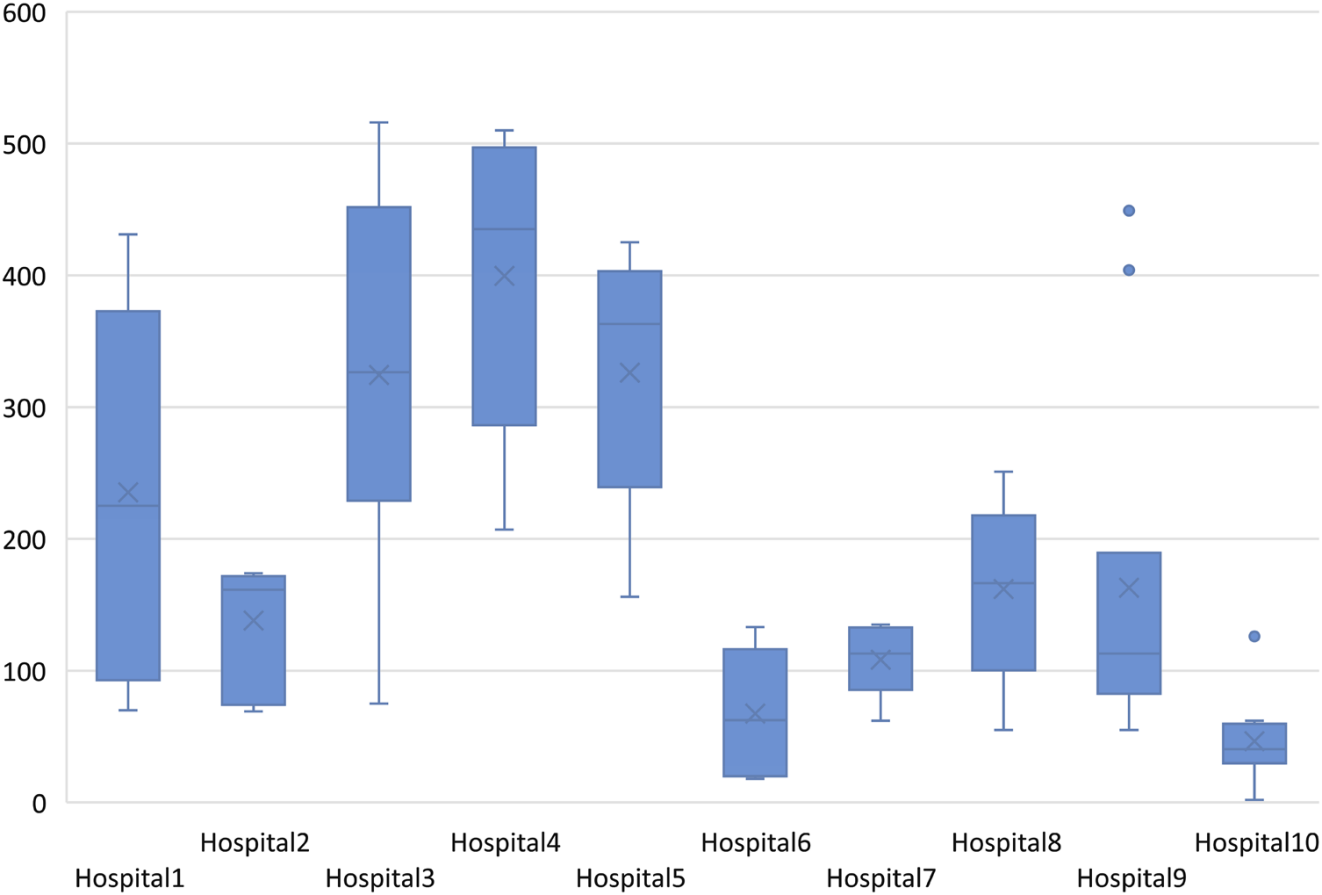
Days between trauma date and registration date in SweTrau. Min = 2 days, Max = 516 days, Median = 139 days

One year (365 days) after the trauma date 84.2% of the patients were registered. Median time from trauma date and registration in SweTrau was just over 4.5 months (139 days, Fig. 4). Some 5.8% (7/120) of the patients were registered within 30 days after the trauma, which is too early for registering the Survival status (30-day mortality) since all seven patients were registered as survivors.

### Comparability

Some 89.6% (43/48 variables) were identical with the Utstein Template of Trauma (*n* = 37) or displayed minor variations (*n* = 6) (Table 2). Five variables were exclusive to SweTrau: *Time of arrival at scene, time of EMS personnel leaving the scene, Type of trauma criteria, Trauma call re-prioritized, Mortality conference.*

## Discussion

This first validation of the Swedish Trauma Registry (SweTrau) confirms that the registry has a high validity, even though there are some areas of improvement that need to be addressed; such as the completion of registration of certain variables. The validated population shows the same properties (approximate median age, predominately male patients, a majority of blunt trauma) as other studies made on trauma populations in Sweden [27, 28] which indicates a representative sample of patients. In the vast majority of cases where an adjustment of NISS score was made at re-registration, this did not lead to the patient being considered severely injured, i.e. no change in morbidity in a significant way. Only three patients (2.5%) had a modification in NISS that actually affected the limit of NISS < 15 < (two altered to > 15 and one to < 15) which is a better result than reported in a Norwegian study [5] (5.6% had a change that affected the ISS < 15 <). This indicates that a cut-off of NISS > 15 for severely injured patients in SweTrau is valid. Although overall accuracy, correlation, correctness and data completeness showed good results (85.8–89.7%), this still means that roughly ten percent of the data in SweTrau does not match when compared between two independent registrars. This is important to remember when conducting and interpreting research based on SweTrau, in addition to the common limitations with registry data.

### Accuracy, correlation and correctness

We found only one study calculating overall accuracy in a trauma registry, also reporting a good result (94.3% [6], compared to 85.8% in our study). Survival status was one of the variables in our study with very high accuracy (99%) and excellent correlation (0.8), which is in concordance with a Dutch study (accuracy 99%, correlation 0.82 [18]). Regarding overall correctness, we identified two additional studies from regional trauma registries that had good (> 85%) correctness as well: 98% [23] (Spanish study evaluating 42 variables) and 97.1% [6] (Finnish study evaluating 32 variables), in relation to 89.7% in our study (evaluating 48 variables and four groups of variables). When the re-registration was made, we found that some data (systolic blood pressure for example) had been rounded in the original registration, which possibly could explain some discrepancies in accuracy and correlation. This is taken into consideration when calculating correctness. Among the seven variables that had poor accuracy, four also showed poor correctness: *NISS*, *ISS*, *Time until first key emergency intervention* and *Pre-injury ASA physical status classification*. Additionally, *Pre-injury ASA physical status classification* had only moderate correlation, indicating that more instructions for assessing ASA score may be needed. For example, we found that BMI and smoking were judged in different ways when assessing the ASA score. The individual results of NISS and ISS may also seem problematic, however, NISS and ISS performed well when analyzed as *grouped by* < *15* < and *grouped by 1–8, 9–15, 16–24 and 25–75* with both good accuracy and correlation. Since most studies group the patients in some way, we believe that the discrepancies of the individual scores have less clinical relevance. The low performance of accuracy of ISS is also shown in a Dutch study [18] (63%), where the correlation of ISS was slightly lower than in our study (0.84 vs 0.91), although still considered excellent ($\ge$ 0.8), similar to another study from the Netherlands [19] (0.81 vs. 0.91 in our study). The calculation of NISS and ISS is furthermore complicated by the fact that the registrars often have to interpret radiology reports which do not specify the extent of the injury (e.g. should a “minor cerebral contusion” be assessed as “tiny < 1 cm” or “small 1–4 cm”?). This assessment might be more concordant if registrars would have the possibility to perform local validations of a number of patients each year. *Time until first key emergency intervention* may finally be hard to register because of the hospitals’ multiple ways of recording data in patient charts; many times in several different modules that may or may not be linked. A national coherent system of recording in patient charts may improve this.

### Timeliness and case completeness

Ideally, the timeliness should be 31 days post trauma date, however, our study showed a median time from trauma date to registration of approximately 4.5 months. The delay could in isolated cases be due to waiting for post-mortem protocols, although this concerns a very small portion of the patients. This lead us to suggest that more focus should be directed towards improving timeliness in SweTrau so that complete and reliable data could be extracted and interpreted in a timely manner. Of the validated hospitals, only two had registered patients before the mortality variable should be recorded (i.e. too early) which might be improved by more information about the importance of waiting 30 days after the trauma before finalizing the registration.

In the literature, we have found only two studies regarding case completeness, with very diverse results: 97.1% [6] and 60.1% [22], respectively. The case completeness in this study (44.3%) should also be interpreted with caution since the patient sample became too small to properly analyze. It is nevertheless an indication that particularly the lowest level of trauma calls (i.e. Trauma Responses) are not being fully covered by SweTrau since all the missing patients consisted of Trauma Responses, making the case completeness for patients with NISS > 15 100%. This suggest that analysis of severely injured patients in SweTrau is reliable while the cohort of patients with minor injuries might not be representative, possible due to not being prioritized during registration.

Because case completeness is dependent on the registry´s inclusion criteria (in SweTrau “all trauma calls”) this also relies on finding all Trauma Responses, instead of only Trauma Alerts and patients with NISS > 15. Today, SweTrau has adopted a way to approximate case completeness by comparing its registered cases with the Swedish Intensive Care Registry, which is possible due to the unique personal identification number that everyone in Sweden have. This, however, focuses mainly on finding patients with NISS > 15 and Trauma Alerts and is tainted with several problems (not all intensive care wards are affiliated, a trauma patient can have several different registrations for the same trauma etc.). A way to increase this precision would be to simultaneously compare the patients with The Cause of Death Registry, as well as pinpointing a certain number of trauma diagnoses and compare these patients with the Patient Registry, where all diagnoses in Sweden are registered, but there is no absolute correlation between a certain ICD-10, chapter 19, (S00-T98) diagnosis and the inclusion criteria for SweTrau. Also, the issue with finding all Trauma Responses remains to improve case completeness. We found this to be virtually impossible without comparing the emergency ledgers at each individual hospital, as is done in this study.

### Data completeness

Data completeness of SweTrau seems to be in line with the few studies that exists: slightly lower than in two regional trauma center studies (88.5% vs 93.4% [6] and 92.8% [23]) but higher than in a large study by Ringdal et al. (81.3% of the variables in our study had > 80% data completeness compared to 78% in the multicenter study [20]). *RR clinical category upon arrival of EMS personnel at scene, RR clinical category upon arrival in hospital* and *SBP clinical category upon arrival of EMS personnel at scene* all had poor data completeness (< 70%). The clinical category is an estimation which is used when there is no specific value recorded (for example, respiratory rate (RR) can be “normal”, “fast”, “slow”, “gasp” or “no respiration”). However, this is not always noted on the patient chart which could be a reason, we believe, for missing data. Making these parameters mandatory before signing the patient charts could improve compliance. Blood tests, such as *Base excess, Time until normal base excess* and *Coagulation: INR* also exhibited poor data completeness. Some of these missing values are unavoidable since not all trauma patients (especially not Trauma Responses) get blood tests taken. Moreover, the issue with missing data on *Base excess* and *Time until normal base excess,* as well as pre-hospital recordings on RR and SBP, has also been noted in an international multicenter study by Ringdal et al. [20]. Lastly, *Time of trauma* was missing in almost 50% of the cases—somewhat understandable since the time of trauma in many cases is unknown and this variable should consequently be judged with caution.

### Comparability

The minor variations of the Utstein Template of Trauma variables consisted of recording *time of alarm* and *time of arrival in hospital* instead of *time from alarm to hospital arrival*, as well as recording *time of trauma* and *date of discharge* instead of *length of stay*. Moreover, SweTrau uses *either* venous *or* arterial blood gas for base excess, while the Utstein Template states arterial blood gas—a disparity which should not affect the result in a significant way. In summary; with the vast majority of the variables being consistent with the Utstein Template of Trauma, we deem that the data in SweTrau is comparable to other trauma registries using the Utstein Template of Trauma.

### Limitations and strengths

Due to the COVID-19 pandemic the examined patient population became considerably smaller than intended, which is a major limitation of this study. Despite several attempts, we were not allowed to visit several hospitals due to visitor restrictions, and exemptions were not made since validation was not considered a priority under the circumstances. The loss of hospitals was, however, random and we therefore still consider the population to be representative, although the sample size is less than desired. One of the strengths of this study is that the data is from 2018 which therefore reflects the actual situation for the trauma registrars before the additionally cut-down in time and resources following the outbreak of the COVID-19 pandemic. Another strength is that we have validated each case on-site at the actual hospital—which certainly is a very time consuming way to validate but as a result gave us the exact same prerequisites as the local registrars.

## Conclusion

This study shows that the overall data validity of SweTrau is high; with good accuracy (85.8%), correctness (89.7%) and data completeness (88.5%), as well as strong or excellent correlation (87.5%). The data in SweTrau is comparable to other trauma registries that use the Utstein Template of Trauma. Areas of improvement are, however, timeliness and case completeness. The authors would like to recommend SweTrau to continuously perform regular validations on-site in the future.

## Data Availability

Data available from the authors upon reasonable request.
